# The role of G protein-coupled receptor in neutrophil dysfunction during sepsis-induced acute respiratory distress syndrome

**DOI:** 10.3389/fimmu.2023.1112196

**Published:** 2023-02-20

**Authors:** Yi Wang, Cheng-long Zhu, Peng Li, Qiang Liu, Hui-ru Li, Chang-meng Yu, Xiao-ming Deng, Jia-feng Wang

**Affiliations:** ^1^ Faculty of Anesthesiology, Changhai Hospital, Naval Medical University, Shanghai, China; ^2^ Jiangsu Province Key Laboratory of Anesthesiology, Xuzhou Medical University, Xuzhou, Jiangsu, China; ^3^ Faculty of Anesthesiology, Weifang Medical University, Weifang, Shandong, China

**Keywords:** sepsis, ARDS, neutrophil, GPCR, desensitization, internalization

## Abstract

Sepsis is defined as a life-threatening dysfunction due to a dysregulated host response to infection. It is a common and complex syndrome and is the leading cause of death in intensive care units. The lungs are most vulnerable to the challenge of sepsis, and the incidence of respiratory dysfunction has been reported to be up to 70%, in which neutrophils play a major role. Neutrophils are the first line of defense against infection, and they are regarded as the most responsive cells in sepsis. Normally, neutrophils recognize chemokines including the bacterial product N-formyl-methionyl-leucyl-phenylalanine (fMLP), complement 5a (C5a), and lipid molecules Leukotriene B4 (LTB4) and C-X-C motif chemokine ligand 8 (CXCL8), and enter the site of infection through mobilization, rolling, adhesion, migration, and chemotaxis. However, numerous studies have confirmed that despite the high levels of chemokines in septic patients and mice at the site of infection, the neutrophils cannot migrate to the proper target location, but instead they accumulate in the lungs, releasing histones, DNA, and proteases that mediate tissue damage and induce acute respiratory distress syndrome (ARDS). This is closely related to impaired neutrophil migration in sepsis, but the mechanism involved is still unclear. Many studies have shown that chemokine receptor dysregulation is an important cause of impaired neutrophil migration, and the vast majority of these chemokine receptors belong to the G protein-coupled receptors (GPCRs). In this review, we summarize the signaling pathways by which neutrophil GPCR regulates chemotaxis and the mechanisms by which abnormal GPCR function in sepsis leads to impaired neutrophil chemotaxis, which can further cause ARDS. Several potential targets for intervention are proposed to improve neutrophil chemotaxis, and we hope that this review may provide insights for clinical practitioners.

## Introduction

Sepsis is a severe life-threatening inflammatory response syndrome secondary to an infection, which is often characterized by multiple organ dysfunction (1, 2). The treatment of sepsis is still dominated by antibiotic therapy, respiratory support, fluid therapy, and organ function support since there are no approved specific drugs for sepsis currently (3). This kind of non-specific treatment prolongs the survival time of sepsis patients, and sepsis may develop into a critical illness lasting several weeks or months, further increasing the financial burden on patients’ families (4). Even with the use of these extrinsic supports, sepsis survivors often have other complications such as cognitive dysfunction, immune dysfunction, and neuromuscular disorders (5). It remains the leading cause of death in hospitalized patients, resulting in a heavy economic burden of medical care. In 2016, a systematic review and meta-analysis estimated that there are 31.5 million sepsis cases worldwide each year, with potentially 5.3 million deaths annually, based on data from seven high-income countries on four continents over the past decade (6). Given the lack of epidemiological studies on sepsis in low- and middle-income countries (LMICs), the actual global cumulative incidence may be higher. In 2020, based on data obtained in more LMICs from 1990 to 2017, an institute estimated that there are 48.9 million sepsis cases worldwide each year, with 11 million sepsis-related deaths annually, accounting for 19.7% of global deaths (7). According to the same study, approximately 50 percent of sepsis cases occurred in children and adolescents (7). Similarly, a recent international multicenter prospective observational study found that neonatal sepsis is the leading cause of neonatal death in LMICs (8). Therefore, sepsis is not only a fatal global syndrome but also a serious public health issue.

The third international consensus definition of Sepsis 3 defines sepsis as a life-threatening organ dysfunction caused by the host’s dysregulated response to infection, which distinguishes sepsis from the infection itself (1). It lies not only in the existence of infections caused by bacteria, fungi, viruses, or parasites but also in organ dysfunction caused by the dysfunctional response of the host to infections. In sepsis caused by secondary infection, the host recognizes microbial-derived pathogen-related molecular patterns (PAMPs) or endogenous damage-related molecular patterns (DAMPs) through a series of pattern recognition receptors (PRRs) at the cell membrane or in the cells, thereby activating innate immune cells to produce cytokines that mediate downstream signaling pathways (9–11). In the development of sepsis, the immune system is over-activated initially, characterized by overstimulated neutrophils and overload of the inflammatory cascade, and followed by immunosuppression, as shown by neutrophil dysfunction and increased apoptosis of lymphocytes (11–13). The phenomenon of early immune overactivation and late immune suppression in sepsis is called immune imbalance. As the most abundant innate immune cells at the locus of infection, neutrophils are the initial defense line of the host against infection and play a crucial role in eliminating local infection and injury healing (1). This cytokine storm is one of the important reasons why sepsis is difficult to control and the mortality remains high.

As a normal response to infection, neutrophils recognize chemokines including the bacterial product N-formyl-methionyl-leucyl-phenylalanine (fMLP), complement 5a (C5a), and lipid molecules Leukotriene B4 (LTB4) and C-X-C motif chemokine ligand 8 (CXCL8), and then enter the site of infection through mobilization, rolling, adhesion, migration, and chemotaxis (14). However, sepsis may induce dysfunction in neutrophil chemotaxis, characterized as compromised migration of neutrophils targeting infected organs (15), while a large number of neutrophils accumulate in the lung. The inflammatory mediators released by neutrophils will trigger an overwhelming cascade of inflammatory responses, further exacerbating the activation of other innate immune cells (16), leading to severe acute lung injury (ALI) and even acute respiratory distress syndrome (ARDS) (4). In non-sepsis conditions, neutrophils in the lung may even undergo reverse migration, returning to the vasculature after local infiltration at the site of inflammation (17). They will then return to the bone marrow to undergo apoptosis *via* C-X-C motif chemokine receptor 4 (CXCR4) reacts to CXCL12 (stromal cell-derived factor-1, SDF-1) (18). But in sepsis, neutrophils will be seized in the lungs and their apoptosis processes are significantly inhibited (18). Experiments using a murine model of abdominal infection showed that the reversing migration of neutrophils in the blood circulation produces excessive inducible Nitric Oxide Synthase (iNOS) and neutrophil extracellular traps (NETs), and promotes tissue inflammation and damage, which is positively correlated with the degree of lung injury (19–21).

In recent years, although some progress has been made in the identification and management of sepsis in terms of microbial pathogenicity and host reactivity, the physiological and pathological mechanisms leading to sepsis-induced lung injury are still not fully elucidated. Previous studies have shown that neutrophil migration dysfunction is associated with sepsis prognosis (22–24), providing a new insight that targeting the disturbance in neutrophil chemotaxis might be promising in reversing sepsis-induced organ dysfunction (25, 26), and the receptors responsible for chemotaxis might be important therapeutic targets in sepsis (27). It has been reported that 18 out of 23 human chemokine receptors belong to G protein-coupled receptors (GPCRs), whose expression and downstream signaling pathways are precisely regulated (28). The typical function of these chemokine receptors is to coordinate cell polarization and induce the directed migration of leukocytes to their chemokine ligands (29, 30). Furthermore, neutrophils in higher vertebrates respond to more than 30 GPCR signals corresponding to a range of chemotactic agents that affect cell polarization and thus migration in tissues (31). As the most abundant and diverse group of eukaryotic cell surface receptors, with more than 800 forms of human expression (32, 33), GPCRs expressed on various cell surfaces mediate neutrophils to monitor the danger signals generated by various external stimuli such as hormones, pathogens, inflammatory factors, and control their chemotaxis to infection foci to play an immune role (34). Approximately 34% of drugs approved by the US Food and Drug Administration (FDA) are GPCR-related (35), and these receptors are the largest family of pharmaceutically available proteins in the human genome (36), demonstrating their importance as key therapeutic targets. The purpose of this review is to elucidate the role of GPCRs in neutrophil dysfunction during sepsis-induced acute respiratory distress syndrome (ARDS) to provide therapeutic targets for the corresponding clinical treatment.

## The role of neutrophils in health

**Figure 1:**
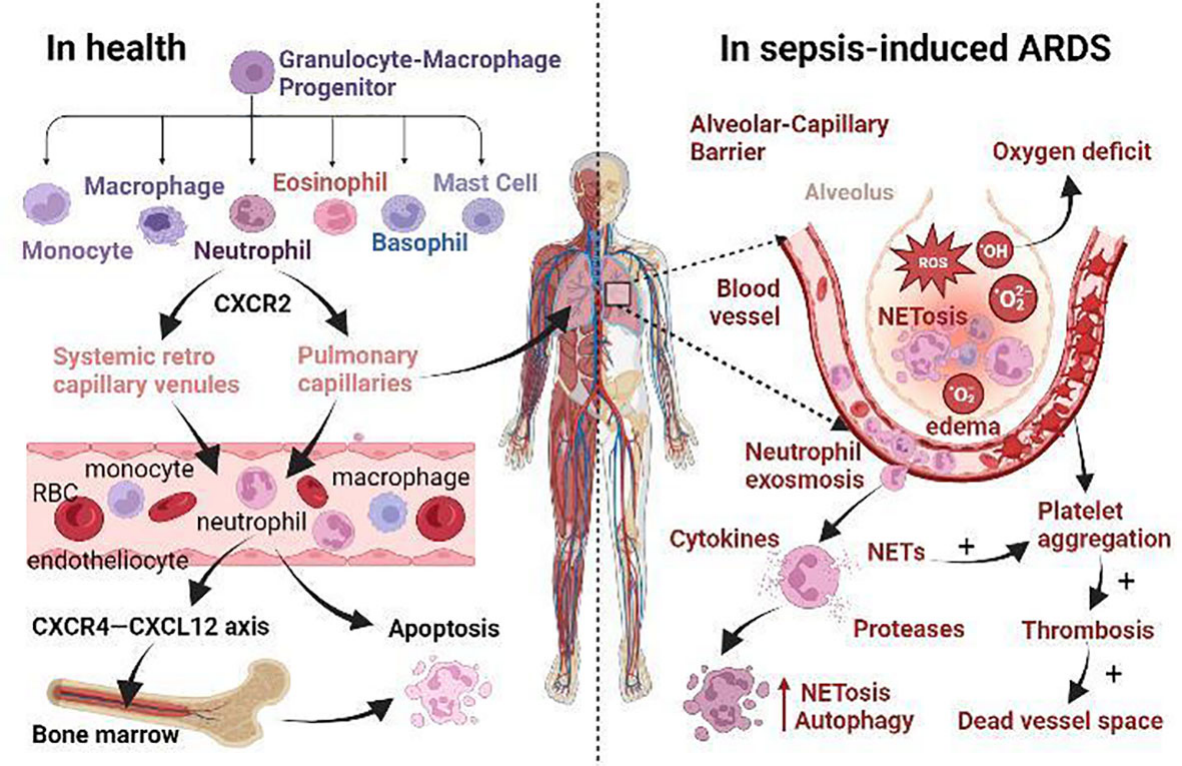
The role of neutrophils in health and the mechanism of sepsis-induced ARDS. (i) Normally, neutrophils originate from granulocyte-macrophage progenitor (GMP) in the bone marrow. When neutrophils mature, CXCR2 promotes their release from the bone marrow. Then they enter the systemic circulation *via* systemic retro capillary venules and into the pulmonary circulation *via* pulmonary capillaries. Finally, they enter the liver, spleen and other reticuloendothelial organs, or even return to the bone marrow *via* CXCR4-CXCL12 axis for apoptosis. (ii) In sepsis-induced ARDS, neutrophil apoptosis is delayed and migration is impaired, and a large number of neutrophils accumulate in the lungs Neutrophils promote self-aggregation and platelet aggregation by releasing ROS, NETs, protease, and other substances, leading to pulmonary ischemia and hypoxia, tissue edema, and micro thrombosis. Prolonged retention of late apoptotic neutrophils in pulmonary capillaries leads to the formation of a vascular dead lumen. Intense inflammatory response, endothelial barrier breakdown, alveolar edema, neutrophil dysfunction, and microcirculation disturbance are the main pathological changes of septic ARDS.

Neutrophils, as the main effector cells of innate immunity and involved in the initiation, diffusion, and resolution of inflammation, account for 50~70% of all circulating white blood cells (37). They are generally regarded as the prototype of fast amoeboid-migrating cells, which means they have plastic deformability that enables them to take distinct migration strategies to move optimally in different microenvironments (17, 38). Normally, neutrophils reside in three different pools, known as the proliferative, circulating, and marginating pools (39). The number of neutrophils in each pool is affected by individual health state and the maturational development of cells. The proliferative or mitotic pool is composed of early neutrophil precursors such as myeloblasts, promyelocytes, and young myelocytes, which are located in the bone marrow and replenish the neutrophil population through mitosis (40). An adult produces about 10^11^ neutrophils per day, with an estimated 10^9^ cells/kg of body weight leaving the bone marrow (41–43). Granulocyte colony-stimulating factor (G-CSF) and granulocyte-macrophage colony-stimulating factor (GM-CSF) have the potential to promote motivation, maturation, and activation of them (44), and to extend their lifespan (45). The release of neutrophils from the bone marrow is positively regulated by CXCR2 signaling and negatively regulated by the CXCR4-CXCL12 axis (**Figure 1**), keeping neutrophils in balance in the circulating and marginating pools (46). To avoid altering the activity of neutrophils *in vitro*, recent *in vivo* isotope or nanotechnology labeling experiments have shown that neutrophils are normally short-lived, with the mean half-life of inactive neutrophils ranging from 7 to 12 hours (47–50). Specifically, human neutrophils originate from granulocyte-macrophage progenitor (GMP) cells, which produce neutrophil promyelocytes that proliferate and differentiate into myelocytes (51–54). Following the myelocyte stage, the neutrophil progenitors lose their capacity to divide and take 4-6 days to mature (49, 55). Under noninfectious conditions, mature neutrophils are released into intravascular circulating pools and finally enter the marginating pools of reticuloendothelial organs such as the liver, spleen, and lung, or even return to the bone marrow for apoptosis (56, 57). Apoptotic neutrophils are phagocytosed by macrophages to limit inflammatory responses (58).

## The role of neutrophils in general infection

During general infection, neutrophils in the circulating blood must first adhere to the blood vessel walls before being recruited to the infection site in response to the chemokine gradient. The upregulation of endothelial adhesion molecules and the loosening of vascular tight connections facilitate the migration of neutrophils to the target tissue (11). Activated endothelial cells upregulated the prestored P-selectin (CD62P) from Weibel-Palade bodies within minutes and the *de novo* synthesized E-selectin (CD62E) within 90 minutes (59, 60). These two selectins on endothelial cells bind to glycosylated ligands such as CD44, E-selectin ligand 1 (ESL1), and p-selectin glycoprotein ligand 1 (PSGL1) on neutrophils (61), trapping endovascular free-flowing neutrophils to the surface of endothelial cells, where they roll along the blood vessels toward the bloodstream. Rolling neutrophils recognize CD34, PSGL1, and glycosylation-dependent cell adhesion molecule (GlyCAM) on the surface of endothelial cells *via* L-selectin (CD62L) to be captured by endothelial cells again (62, 63). Bacterial components and pro-inflammatory cytokines promote L-selectin shedding but increase β2-integrins expression on neutrophils such as LFA-1 (lymphocyte function-associated antigen 1, CD11a/CD18) and MAC1 (CD11b/CD18) (64). These β2-integrins adhere to endothelial cells by binding to intercellular adhesion molecule-1 (ICAM-1) and vascular cell adhesion molecule-1 (VCAM-1) with high affinity (65). Therefore, it has been suggested that the migration of neutrophils depends more on integrins than selectin (63). However, both selectin and integrin-mediated adhesion are brief and weak, promoting neutrophils to roll along endothelial cells and inducing their exosmosis to inflamed sites (66, 67).

Subsequently, neutrophils respond to bacterial toxins such as lipopolysaccharide (LPS) carried by the infectious agent, bacterial products such as fMLP, and chemokines produced by immune system activation such as C5a, LTB4, platelet activator (PAF), and interleukin (IL) -8 (CXCL8), all of which are effective activators of neutrophils (68–70). They are usually positively charged molecules that bind to negatively charged heparan sulfates and thus anchor to endothelial cells to form a certain intravascular chemotactic gradient (71). The rolling of neutrophils helps them to contact chemokine-decorate endothelial cells, thereby inducing neutrophilic activation. These chemo-attractants also induce the re-localization of integrins such as MAC1 stored intracellularly to the surface of neutrophils (72). The cytoskeletal protein talin1 binds to the β subunit of the activated integrin cytoplasmic tail to induce the extension of LFA1, thereby reducing the affinity of neutrophils to endothelial cells through conformational changes and promoting the slow rolling of neutrophils on endothelial cells (14). Another protein containing the FERM domain, kindlin 3 (also known as fermitin family homologue 3), binds to the same site on integrin and induces LFA1 to adopt a high-affinity conformation that promotes neutrophil stasis on endothelial cells (73). Due to fluid shear stress, endothelial cell structures are more elongated in the direction of blood flow, and neutrophils tend to crawl vertically at the endothelium-cell junctions (74). Neutrophils preferentially select endothelial tricellular corners with fewer junctional proteins and less orderly arrangement for paracellular migration (between endothelial cells) (75) over less efficient transcellular migration (through endothelial cells) (76).

When they finally migrate to the source of infection or damaged tissue, neutrophils emit pseudopods through membrane invaginations that envelop cytotoxic proteins, peptides, and enzymes in the phagosomes (40). The formation of the phagosomes attracts neutrophilic granulocyte particles to bind to them and degranulate themselves (77). At the same time, NADPH oxidases located on the phagosome membrane are activated to produce superoxide anion (O_2_^-^) and metabolize into hydrogen peroxide (H_2_O_2_) and other reactive oxygen species (ROS) (27). ROS, in turn, induce NETs release from activated neutrophils, which trap and kill pathogenic microorganisms to prevent their spread. They are a network of chromatin fibers that consist of DNA, citrullinated histone 3 (Cit-H3), myeloperoxidase (MPO), neutrophil elastase (NE), cathepsin G (CatG), proteinase-3 (PR-3), and other granular proteins (78). When the NETs formation is associated with cell death, neutrophils undergo suicidal dissolution of NETosis, a process that may release inflammatory mediators (79). Specifically, NETosis is a programmed neutrophil death distinct from apoptosis or necroptosis that facilitates host defense against pathogens and is characterized by nuclear delobulation, histone citrullination, chromatin decondensation, membrane permeabilization, and NETs release (80, 81). It was first described in 2004, where NET release and cell death were observed in response to high doses of phorbol-12-myristate-13-acetate (PMA) (82).

To date, three forms of NETosis are known, including suicidal, noncanonical, and vital NETosis (83). Suicidal NETosis mediated ROS production, MPO activation, and cytoplasmic release of NE by NADPH oxidase (83). In the presence of H_2_O_2_, MPO catalyzes the oxidation of chloride to hydrochloric acid and induces neutrophils to release NE, which together mediate chromatin decondensation during NETosis (84). In the cytoplasm, NE binds to the actin skeleton and disintegrates actin by degrading F-actin, thereby inhibiting neutrophil movement (85). In the nucleus, NE cleaves and inactivates histones, resulting in chromatin relaxation and DNA decondensation (84). In addition, peptidyl arginine deiminase 4 (PAD4), activated by high intracellular calcium concentrations induced by ionic carriers or bacterial products, mediates chromatin decondensation by histones citrullination (86, 87). Gasdermin D (GSDMD) is an executor of suicidal and noncanonical NETosis (81, 88). During suicidal NETosis, GSDMD is cleaved by NE and GSDMD-p30 pores are formed in the nuclear, granular, and plasma membranes (81). In turn, the breakdown of granules further promotes the release of NE in the cytoplasm, thus further cleaving GSDMD (88). Decondensated chromatin is released extracellularly through GSDMD-p30 pores or GSDMD-driven membrane rupture, ultimately resulting in neutrophil death (82, 88). Noncanonical NETosis is a novel suicidal NETosis pathway, which relies on the induction of non-classical inflammasomes to gram-negative bacterial LPS and activates mouse caspase-11 and human caspase-4/5 to cleave GSDMD to produce GSDMD-p30 pores, ultimately resulting in NETs release and cell death (81, 89). At this point, the release of NETs is dependent on Toll-like receptor 4 (TLR4) expression on platelets and P-selectin-mediated platelet-neutrophil interactions (90).

Suicidal and noncanonical NETosis release NETs relatively slowly, taking about 3 hours, while vital NETosis releases NETs only half an hour, and neutrophils that produce vital NETosis do not die immediately but remain phagocytic, migratory, and bactericidal (91). Vital NETosis is stimulated by bacteria, bacterial products, activated platelets, or complement proteins to induce the rapid release of NETs, and does not necessarily rely on NADPH oxidase to induce NE cleavage (87, 92). Moreover, the key features of vital NETosis are rapid histone citrullination, nuclear blebbing, and the vesicular transport of nuclear blebs to the plasma membrane, in which the nuclear membrane is swollen but not ruptured (83). An electron microscopy study showed that these vesicles of Vital NETosis exocytosis on the plasma membrane after budding from the outer nuclear membrane (ONM), thus ensuring the integrity of the nuclear and plasma membrane during NETs release (93). Studies have shown that neutrophil activation is involved in the initiation of NETosis through bacterial toxins or surface receptors. GPCRs (94), tumor necrosis factor (TNF) (95), Fc receptors (94), and TLR4 (96) on neutrophils have been reported to bind to ligands that trigger the release of calcium stored in the endoplasmic reticulum and induce NETosis by activating PAD4 to make histones citrullinated (97, 98). Among several chemokine receptors, CXCR1, CXCR2, and CXCR4 are involved in the formation of NETs (99). On the one hand, CXCR2 induces NETs formation by cooperating with PSGL1 (100). On the other hand, CXCR2 is also involved in neutrophil circadian rhythm regulation, altering the ability of NETs formation by disarming processes involving the neutrophil proteome (101). This circadian pattern is characterized by functional and phenotypic changes from neutrophil release to clearance, and this process is described as neutrophil aging (102). CXCR4 acts by releasing macrophage migration inhibitory factor (MIF), which in turn leads to NETs formation (103).

Additionally, the migration of activated neutrophils also depends on cytoskeletal dynamics mediated by molecular motor (myosin, kinesin, and dynein) drives (104, 105). Myosin is mainly involved in vesicle transport on actin filaments, while kinesin and dynein contribute to transport along microtubules. Kinesin moves intracellular cargo toward the plus-ends of the microtubules, while dynein moves toward the minus-ends of them (106). Inflammatory chemokines can act on intracellular signaling pathways through corresponding receptors (mostly GPCRs) to induce neutrophils to migrate by a polymeric contractile balance between anterior branch actin and posterior myosin II-dependent actin (107–110). Studies of zebrafish larvae have presented cytoskeletal requirements for neutrophil redirection (111, 112) Firstly, actin-related protein 2/3 (Arp 2/3)-mediated dendritic actin networks determine the extensive search phase of neutrophils, followed by rapid actin flow that accelerates cell migration to the source of infection. Formyl peptide receptors (FPRs) are G-protein-coupled receptors that are expressed on the surface of neutrophils that recognize peptides containing N-formylated methionine, such as fMLP. FPRs transmit chemotactic signals that mediate host defense and inflammatory responses such as cell adhesion, directed migration, particle release, and superoxide production. The human FPR family consists of three members, in which FPR3 is expressed in monocytes but only FPR1 and FPR2 are expressed in neutrophils (**Table 1**) (113). Notably, a variety of non-GPCRS receptors are expressed on the surface of neutrophils, including cytokine receptors, integrins, Fc-receptors, and other innate immune receptors (**Table 1**), which are also critical for the differentiation, adhesion, recruitment, and phagocytosis of neutrophils (114).

**Table 1:** Common receptors and their corresponding chemo-attractants and GRKs.

Receptor	Function	Chemoattractant	GRK
Chemokine receptors			
CXCR1	Neutrophil recruitment	CXCL1-8	GRK2,6
CXCR2	Neutrophil activation and recruitment	CXCL1-8	GRK2, 5, 6
CXCR4	Bone marrow homing	CXCL12	GRK2, 3, 6
CCR7	Neutrophil recruitment	CCL19, CCL21	GRK3, 6
CCR9	Neutrophil recruitment	CCL25	GRK2
Chemoattractant receptors			
C3aR	Inhibition of neutrophil mobilization	C3a	
C5aR, C5L2	Neutrophil recruitment	C5a	GRK2
BLT1	Neutrophil swarming and recruitment	LTB4	GRK6
FPR1, FPR2	Neutrophil activation, adhesion, and recruitment	Bacterial and mitochondrial formylated peptides, e.g., fMLP	
Atypical chemokine receptors			
ACKR1	Chemokine aggregation	CC chemokines	
ACKR2	Chemokine scavenger receptor	CC chemokines	
ACKR3	Opioid receptor	Opioid peptide	GRK2, 5
ACKR4	Chemokine scavenger receptor	CC chemokines	GRK3

CXCRs, C-X-C chemokine receptors; CXCLs, C-X-C chemokine ligands; CCRs, C-C receptors; C3aR, complement factor 3a receptors; C3a, complement factor 3a; C5aR, complement factor 5a receptors; C5L2, complement factor 5a receptors 2; C5a, complement factor 5a; BLT1, leukotriene B4 receptors; LTB4, leukotriene B4; FPRs, formyl-peptide receptors; fLMP, formyl-methionyl-leucyl-phenylalanine; ACKRs, atypical chemokine receptors; GRKs, G-protein-coupled receptor kinases.

## The contribution of neutrophils and GPCRs in sepsis

It is well-accepted that neutrophil dysfunction occurs in sepsis and has been considered the main cause of organ failure (27, 115–117). Neutrophil degranulation and endothelial dysfunction are shown to be the core events of the pathophysiology of sepsis (77, 118). In the early stage of sepsis, mature neutrophils in the bone marrow are rapidly released, increasing 10-fold in circulation within hours, and largely accumulate in the lungs, according to autopsy reports (119). Bacterial products and over-release of pro-inflammatory cytokines such as TNF-α, IL-1b, IL-6, and IL-17 increase G-CSF expression and indirectly mobilize neutrophils by altering the balance between CXCR4 and CXCR2 ligands in bone marrow, affecting their release, activation, and migration (120, 121). During sepsis, the spontaneous apoptosis of neutrophils is delayed, but other types of death such as necrosis, necroptosis, pyroptosis, NETosis, and autophagy may happen (10, 116, 122). It is well established that the proportion of neutrophil NETosis (123) and autophagy (124) increases in sepsis with reduced apoptosis (125), but the other three types of neutrophil death remain poorly understood (**Figure 1**). A study showed that 50% of neutrophils were apoptotic after 24 hours of *in vitro* culture, compared with only 5-10% in sepsis (125). In models of ALI caused by cecal ligation puncture (CLP) or endotoxemia, the proportion of lung neutrophils undergoing apoptosis within 24 hours was significantly reduced (126). Moreover, septic animals and patients showed a reduction in neutrophil migration (127, 128). Due to migration dysfunction, neutrophils with delayed apoptosis cannot effectively reach the site of infection to eliminate pathogenic bacteria and their phagocytic activity is decreased. Instead, most of them are detained in the lung, liver, and other organs, mediating nonspecific organ damage through the release of ROS, NETs, protease, and other cytotoxic substances (129–131).

In sepsis, the complement system is activated, releasing small fragments such as C3a and C5a, which have potent pro-inflammatory effects (132). C5a binds to its receptors (C5aR and C5L2) on the surface of activated neutrophils to promote the release of NETs, in which DNA fibers and histone (H3 and H4) networks provide scaffolds for the aggregation, relocation, and activation of platelets, neutrophils, and red blood cells (133). TLR4-mediated platelet activation increases endothelial cell adhesion at the site of inflammation (66) and promotes neutrophil activation, exosmosis, and aggregation by expressing higher levels of P-selectin (**Figure 1**) (134). Platelet-derived chemokine heterodimers (of CXCL4 and CXCL5) are also important for neutrophil recruitment (135), and endothelial presentation of CXCL5 depends on platelets (136). Activated platelets interact with neutrophils through β_2_-integrin LFA-1, thereby enhancing neutrophilic activation and lowering the threshold for NETs release (137, 138). Interactions between activated endothelial cells further promote the release of NETs (**Figure 1**) (139–141). Many components of NETs, such as DNA, histones, and granular proteins, all have procoagulant activity (142). DNA initiates the intrinsic clotting cascade and nucleic acid enhances serine protease thrombin activity (143). Histones promote thrombin production by inhibiting anticoagulants such as antithrombin (AT) and activating protein C (APC) (144). Granular proteins, especially neutrophil elastase, promote thrombosis by inhibiting tissue factor pathway inhibitors (TFPI) and anticoagulants (145). At the same time, coagulation factors, thrombin, plasmin, and APC in the blood induce downstream signaling pathway transduction by activating protease-activating receptors (PARs, a small family of GPCRs) expressed on platelets, endothelial cells, and vascular smooth muscle cells (VSMC) (118), further aggravating tissue and organ damage. There are currently four types of PARs in the human genome, among which platelet expresses PAR1 and PAR4 (146), endothelial cells express PAR1, PAR2, and PAR4 (147), VSMC expresses PAR1 and PAR2 (148, 149), and PAR3 is mainly expressed in bone marrow (150). Of the four major Gα subfamilies, PAR1 and PAR2 mediate signaling *via* Gq, Gi, and G12/13, PAR3 *via* Gq-mediated signaling, and PAR4 *via* Gq and G12/13 (151). In sepsis, these reactions lead to increased vascular permeability, endothelial barrier breakdown, abnormal accumulation of neutrophils, and excessive inflammatory responses, which further aggravate ischemia, hypoxia, tissue hypoperfusion, and microcirculation disorders, eventually leading to organ failure, shock, and even death (152, 153).

## The contribution of neutrophils and GPCRs in sepsis-induced ARDS

Among all the damaged organs, the lung is the first and most frequent organ to be compromised, and ARDS is one of the key prognostic factors for the mortality of septic patients (154). In systemic circulation, neutrophils enter the tissue through systemic retro capillary venules, but in pulmonary circulation, neutrophils migrate through pulmonary capillaries (**Figure 1**) (155, 156). Neutrophils must undergo deformation to pass through the pulmonary capillaries because of their larger diameter, a time-consuming process called neutrophil sequestration, which was first described in 1993 and has been observed using macroscopic radiolabeling imaging devices (157, 158). In contrast to the doughnut-shaped nuclei of mice, human neutrophils are leafy, increasing the flexibility of mature neutrophils to navigate tissue space (159, 160). Nevertheless, the physiological and pathological mechanism of how neutrophil sequestration in sepsis causes ARDS is still poorly understood (161, 162). An uncontrolled immune inflammatory response caused by sepsis eventually leads to multiple organ dysfunction syndromes (MODS) of the heart, brain, liver, lung, kidney, etc (63, 153). Autopsy of these patients with MODS in sepsis revealed abundant neutrophils in the kidneys and lungs (163). It has been found that the functional capillary ratio of the pulmonary microcirculation is decreased in sepsis-induced ALI (164), which represents the occurrence of abnormal tissue perfusion, and organ dysfunction may be caused solely by neutrophils sequestered in the microvascular system (165). In the early stage of sepsis, neutrophils are activated and migrate in the pulmonary capillaries. In the late stage, neutrophils with delayed apoptosis are trapped in the pulmonary capillaries for a long time, resulting in the formation of a vascular dead lumen (**Figure 1**), which further triggers the aggregation of neutrophils and leads to microcirculation disturbance, exacerbating the hypoxia caused by sepsis-induces ARDS (164). This suggests that neutrophil sequestration may be a critical stage in the initiation of multiple organ failure (166) and that damage in one organ by a large accumulation of neutrophils may trigger the same aggregation effect in other organs (167).

In addition, the severity of septic ARDS is positively correlated with the degree of neutrophil infiltration and the intensity of its derived proteolytic enzymes in bronchoalveolar lavage fluid (168). Results from the bronchoalveolar lavage tests showed that IL-2 concentrations were associated with delayed apoptosis of neutrophils (169), and IL-8 (170), and IL-18 concentrations were associated with poor outcomes in patients (171). IL-8 binds to CXCR1 and CXCR2, which are the high-affinity receptors of CXCL. But only CXCR2 rather than CXCR1 is reduced on neutrophils in patients with sepsis, and the interleukin-8 mediated chemotaxis is impaired (172). Xu et al. had shown that an increase in C5a during sepsis inhibits neutrophil IL-8 secretion, resulting in neutrophil migration dysfunction through downstream signaling pathways mediated by C5aR and C5L2 (173). It is concluded that GPCR-mediated aggregation, activation, and apoptosis of the accumulated neutrophils in the lung of septic patients are important causes of ARDS and are related to the release of tissue-destructive immune mediators (174, 175).

## GPCR-mediated positive and negative feedback regulates neutrophil aggregation

During sepsis, the first neutrophils detecting the local tissue infection rapidly release LTB4 within minutes. Other activators such as C5a, LPS, and fMLP, also promote LTB4 release, with a cascade of amplification reactions mediating neutrophils aggregation (176, 177). Neutrophils can sense and monitor the relevant danger signals released by tissues through GPCRs, which promote the release of attractants for communication between cells. The GPCR-mediated positive feedback is the basis of neutrophil aggregation, and its downstream molecular pathways are major triggers of neutrophil polar movement (38).

When GPCR is activated, the Gα subunit of the G protein binds to GTP and dissociates from the Gβγ dimer. The activated Gα induces the generation of a second messenger such as cAMP (178), while the dissociated Gβγ dimer binds to downstream signaling molecules and activates downstream signaling pathways such as phospholipase C β2/3 (PLCβ2/3) and phosphatidylinositol 3-kinase (PI3K) (179, 180). Activation of PLCβ in neutrophils can promote the release of Ca^2+^ from Ca^2+^ pools in the endoplasmic reticulum, leading to an increase in Ca^2+^ concentration and providing power for the secretion of intracellular vesicles or granulosa proteins (40). The human genome encodes four distinct Gα subunits, including Gαs, Gαi, Gαq, and Gα12/13 (181). Gαs activates adenylate cyclase (AC) to catalyze the conversion of ATP to cyclic adenosine monophosphates (cAMP) (182), which is the second messenger that regulates many downstream signaling pathways of GPCRs, while Gαi inversely regulates cAMP concentration by inhibiting AC activity or activating phosphodiesterase (PDE). Gαq activates PLC to hydrolyze phosphatidylinositol 4, 5-diphosphate (PIP2) to inositol triphosphate (IP3) and diacylglycerol (DAG), which acts as a protein kinase C (PKC) activator, while Gα12/13 regulates small GTPases that affect the cytoskeleton of actin and tubulin (183). Despite their differences in function, both the Gαs and Gαi subunits activate the GTPase activity of tubulin to disrupt the stability of microtubules (183). In neutrophils, different Gαi play different roles in GPCR signal transduction, and Gαi does not internalize in response to activation compared to Gαs (184). Gαi2 is responsible for vascular Ca^2+^ flux and neutrophil stasis, while Gαi3 is responsible for neutrophil migration and activation of the PI3K/Akt pathway (185, 186). The helical H5 structure in Gα has been shown to play a key role in mediating the allosteric regulation of GPCRs (187).

Studies have shown that GPCR desensitization is an intrinsic negative feedback mechanism for neutrophils and plays a key role in preventing excessive aggregation of neutrophils to maintain a balance between tissue destruction and host protection (176, 188, 189). Hidalgo and col. showed that neutrophils acquire different phenotypes and functional properties in normal tissues, and they enter these tissues under the action of chemokines, such as CXCR4 signaling to chemotactic neutrophils into the lung in response to CXCL12 stimulation (190, 191). Kinase omics analysis of septic neutrophils revealed impaired activity, indicating an immunosuppressed neutrophilic phenotype (192). C5a down-regulates CXCR4 expression on neutrophils and promotes protease release, which degrades matrix proteins and inhibits the homing effect of CXCL12, ultimately leading to neutrophil phenotype changes (193). At the same time, high levels of C5a may lead to reduced targeted migration of neutrophils (194). However, C5a receptor expression on neutrophils peaks at the first 24 hours after sepsis initiation and gradually declines thereafter (195). PI3K can control the aggregation effect of C5a-mediated neutrophils by regulating the oxidative burst and phagocytosis activity of neutrophils. Inhibition of PI3K *in vitro* up-regulates TLR4-mediated pro-inflammatory cytokine expression in neutrophils (196) and activated TLR4 enhances neutrophils’ chemokine reactivity by down-regulating the expression of G-protein-coupled receptor kinases (GRKs) involved in GPCRs desensitization (197). Therefore, C5a and PI3K interact with TLR during sepsis to upregulate GRKs expression, thereby internalizing and desensitizing GPCRs to reduce neutrophil chemotaxis and negatively regulate neutrophil aggregation. In PI3Kc-/- mice with sepsis, the expression of GRK2 was decreased while that of CXCR2 was increased, and the survival rate was higher, which was consistent with the above conclusion (198). Besides, TLR4-deficient mice did not develop neutrophil migration dysfunction, suggesting that the phenomenon is TLR4-dependent.

## The bias of GPCR expression: G protein or GRK-arrestin pathway

**Figure 2:**
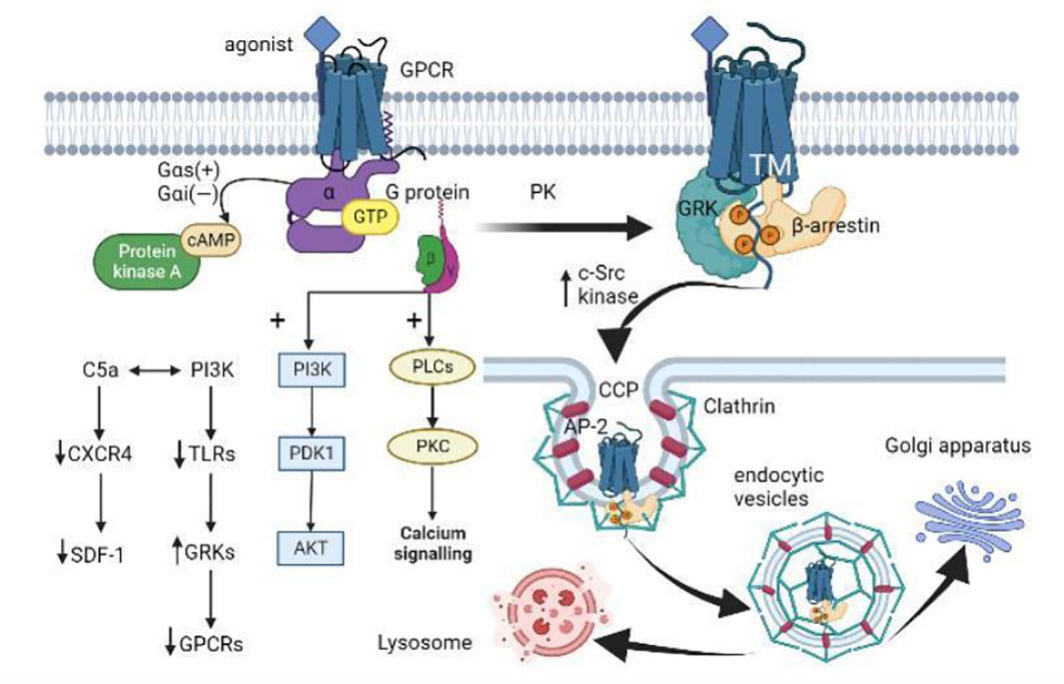
GPCR activation, internalization, and desensitization. (i) When GPCR is activated by an agonist, the Gα subunit of the G protein binds to GTP and dissociates from the Gβγ dimer. Gαs activates adenylate cyclase to catalyze the conversion of ATP to cAMP, while Gαi inversely regulates cAMP concentration. The dissociated Gβγ dimer activates the PLC and PI3K downstream pathways. (ii) GPCR is subsequently phosphorylated by different protein kinases (PKs) like GRK. Then β-arrestin binds to the GPCR-activated transmembrane (TM) core, allosterically modulates its three proline regions (PRs) to induce conformational changes, and spatially impedes the interaction between GPCR and G proteins. β-arrestin induces the aggregation of clathrin and adaptor protein 2 (AP-2) by increasing the activity of c-Src kinases, and targets the receptor on clathrin-coated pits (CCP). (iii) The local membrane invagination promotes the formation of endocytic vesicles. The endocytic vesicles target the Golgi apparatus or lysosomes under the action of Rab proteins and cytoskeleton. (iiii) In addition, activated PI3K promotes GRK activity by down-regulating TLRs expression, thus mediating GPCR desensitization. C5a interacts with PI3K to regulate neutrophil aggregation by down-regulating the expression of CXCR4 in neutrophils and inhibiting the homing effect of SDF-1 (CXCL12).

After GPCR is stimulated by extracellular signals, activated GPCR can induce structural rearrangement of its cytoplasmic region (199) and induce intracellular signal transduction through classical G protein or GRK-arrestin pathway (**Figure 2**) (200, 201), and this signal transduction can be biased in three ways. System bias refers to the differential expression of signaling pathways of the same receptor or ligand in a different time, space, and cell type. Receptor bias refers to the selective action of the same agonist on different receptors to mediate their downstream pathways. Ligand bias refers to the agonist recognized by a single receptor that prefers a certain downstream pathway, which is generally thought to be related to the conformation of the GPCR-arrestin complex (114, 202, 203). Residues near the GPCR binding site are much less conserved than critical residues on the intracellular membrane of the cells, resulting in complexes that have both “clingy” and “hanging” conformations, with the former critical for desensitization of G protein signaling and the latter promoting G protein activation or G protein independent signaling (204, 205). In most cases, G protein signaling is the cause of adverse drug reactions, and ligand bias can be used to reduce such adverse reactions, leading to the development of more promising and safer drugs (206, 207). How these ligands reconfigure cytoplasmic regions of the GPCR to selectively promote G protein, GRK, or arrestin binding is not yet known.

There are more than 286 GPCR structures that can combine with G proteins (208), but only five of them can bind to arrestin and activate GPCR, including two neurotensin receptor 1 (NTSR1) (209), one M2 muscarinic receptor (M2R) (210), one β1 adrenergic receptor (β1AR) (211), and one V2 vasopressin receptor (V2R) (212). Angiotensin II (Ang II), as a biomarker of sepsis severity, is associated with the progression of septic ARDS (213, 214). Modified angiotensin II type 1 receptor (AT1R) can preferentially stimulate the G-protein-mediated or arrestin-mediated signaling pathway after binding to Ang II, and this model system is often used to study the biased signaling of GPCR (215, 216). The GPCR-arrestin interaction has a shorter duration on receptors such as β1AR, but a longer duration on V2R and AT1R (217). GRK and arrestin are important for regulating the duration and amplitude of GPCR signaling (218). Two of the four arrestin subtypes, β-arrestin 1 and β-arrestin 2, are expressed in almost all cells of vertebrates and interact with different GPCRs on the cell surface to induce their downstream non-classical signaling pathways (219).

The continuous activation of GPCR during inflammation leads to the activation of GRK, which in turn negatively regulates the phosphorylation of C-terminal clusters of GPCR, and then the recruitment of inhibitory protein β-arrestin to bind to these clusters (220). The increased β-arrestin also sometimes binds to the activated transmembrane (TM) core of GPCR, allosterically modulates its proline regions (PRs) (221), causing specific conformational changes, and impeding the interaction between GPCR and G proteins in space, further attenuating GPCR signaling (**Figure 2**). Furthermore, β-arrestins that bind to GPCRs influence the activity of regulatory ubiquitin E3 ligase and a stress-related JNK3 kinase (222–224), but they remained in this active conformation after dissociation from the receptor (225–228). β-arrestin conjugated GPCR to SH3-domain proteins (SH3-CPs) *via* three proline domains (P1, P2, P3), each with different affinity to the receptors and downstream effectors (**Figure 2**). Specifically, phosphoric acid sites 5 on GPCRs were coupled to P1 and P2, phosphoric acid sites 2-3 were coupled to P3, and phosphoric acid binding sites 5 induced conformational changes in regions P1 and P2 through the propagation paths of V40→P114→F87 and P36→V34→F123, respectively (221). The different GPCRs phosphorylation sites regulated by different GRKs lead to different β-arrestin conformations and thus different signal transduction functions, which is known as the “phosphorylation barcode hypothesis” (229–231).

## GPCRs internalization/desensitization: A key factor for neutrophil migration dysfunction

It has been shown that GPCRs desensitization, internalization, and signal transduction are mediated by GRKs and the inhibitory protein β-arrestins (**Figure 2**) (232). There are two forms of GPCRs desensitization, including homologous and heterologous desensitization. Heterologous desensitization means that one GPCR signal can act as desensitizing signal of multiple other GPCR types, causing immune cells to develop resistance to several chemotactic stimuli (38). However, *in vitro* studies have shown that human neutrophils experience homologous desensitization initiated by GRKs, as shown by no response to continuous or repeated stimuli (233, 234). GRKs are key serine/threonine protein kinases in which GRK2-3 and GRK5-6 are commonly expressed in cells and tissues, while GRK1 and GRK7 are localized in the retina and GRK4 is localized mainly in the testis (235). There are four GRKs expressed on neutrophils (GRK2, GRK3, GRK5, GRK6), with different target GPCRs. According to the references, GRK2 binds to CXCR1 (236) and CXCR2 (197, 237, 238), GRK5 to CXCR2 (197), and GRK6 to LTB4R1 (239) and CXCR2 (236). Phosphorylation of GRKs at serine (Ser) or threonine (Thr) residues facilitates the high-affinity binding of GPCR-β-arrestin, which partly explains the desensitization of G protein signal transduction (233). After CXCR4 agonist stimulation, GRK2/3 phosphorylates the distal Ser/Thr locus on the C-tail, while GRK6 phosphorylates more at the proximal Ser/Thr locus (240). In addition, GRK2 also promotes the formation of the GRK2-tubulin complex and tubulin phosphorylation, which may be involved in receptor transport (241). GRK2 also phosphorylates ezrin, a protein involved in cortical actin cross-linking with the plasma membrane (242), which may mediate GPCRs internalization and actin cytoskeletal recombination (243). This difference in the phosphorylation site of GRKs may mediate different signaling pathways, which partly explains the different pharmacodynamics of agonists.

β-arrestin recruited by phosphorylated GPCRs through Clathrin-mediated endocytosis is the main pathway for the internalization of GPCRs, which is important for both receptor recycling and intracellular signal transduction (244). Src are a kind of kinases that contain the SH3 domain and recognize the specific PRs conformation in β-arrestin without recognizing the C-terminal shift of GPCRs (221). The binding of β-arrestin to GPCR can also induce clathrin and adaptor protein 2 (AP-2) aggregation by increasing c-Src kinase activity (245–247). Thus, receptors can be targeted at clathrin-coated pits (CCPs) to make GPCRs locally invaginate to promote ingestion *in vivo* (248). Although AP-2 is not the key to forming the CCPs (249), it can recruit transmembrane cargo and auxiliary proteins *via* a specific motif of clathrin (**Figure 2**) (250). These cargo proteins need to contain specific motifs, such as Y*XX*Φ motifs to be recognized by AP-2 (251). S-nitrosylation of β-arrestin by receptor activation or direct modification promotes GPCRs desensitization and concave endogenization, while β-arrestin lacking such post-translational modification prolongs the signaling time of cAMP, extracellular signal-regulated kinase (ERK), and nitric oxide (NO) (252). In this regard, some scholars believe that the desensitization of GPCRs is not signal shutdown, but signal switching, that is, from G protein signal transduction to G protein independent signal transduction, and this G protein independent signal transduction may be mainly mediated by β-arrestin (253, 254). In addition to mediating the internalization of GPCRs, another key role of β-arrestin is to stimulate mitogen-activated protein kinase (MAPK) cascades. As reported, p38 and ERK induce GRK2 phosphorylation at Ser670 and inhibit GRK2 translocation and the following GPCRs internalization (255), except that p38 promotes monocyte migration while ERK inhibits monocyte migration. The anaplastic lymphoma kinase inhibitor LDK378 inhibits Ser670 phosphorylation on GRK2 by inhibiting p38 activation, resulting in enhanced GRK2 translocation and CXCR2 internalization, thus inhibiting the recruitment of myeloid suppressor cells (MDSCs) to the spleen (11).

Subcellular localization is one of the mechanisms regulating GRKs’ activity and GPCRs’ desensitization (256). Furthermore, the movement of GPCRs in different cellular compartments during internalization occurs in vesicles and is regulated by synergistic interaction with cytoskeletal elements (217). Normally, GPCRs are synthesized in the rough endoplasmic reticulum (ER) and move from the Golgi apparatus to the plasma membrane in the form of vesicles, which is a down-direction transport, while the vesicles formed after the internalization of GPCRs are directed to the lysosome or proteasome, which is a reverse transport (217). Biased agonists stimulate GPCRs in the plasma membrane with a sustained and strong effect, but not in the intracellular compartment, suggesting that the production of second messengers such as Ca^2+^, cAMP, IP3, and DAG is essential for subcellular localization (257). The production of free vesicles is dependent on dynamin, which exists in three subtypes in mammals and binds to acidic phospholipids on the cytoplasmic side of the plasma membrane *via* the pleckstrin homology (PH) domain (**Figure 2**). During the internalization of GPCRs, the dynamin is located at the neck of the budding vesicles and relies on the hydrolysis of GTP to form a helical polymer conformation that promotes the fission of the underlying tubular membrane section, resulting in the generation of endocytic vesicles. This dynamin is also present in the actin reticulum associated with the Arp 2/3 complex (258, 259). Some desensitized neutrophil GPCRs can be reactivated by cytoskeletal destruction (114). Rab proteins are a kind of monomer GTP proteases, which are expressed differently on different membranes and can regulate vesicle docking and fusion. Their activities are partly regulated by the concentration of PI3K (260). A few studies have demonstrated the interaction between GPCRs internalization and various Rab proteins in different vesicular compartments. Among them, Rab5a mainly binds to the last 10 amino acids of AT1AR and participates in the receptor cycle of reverse transport (261), while Rab11 is associated with the circulation of receptors that are transported downstream and is located in the trans-Golgi network (TGN), post-Golgi vesicles, and recycling endosomes (262, 263). This suggests that GPCRs can regulate the activity of intracellular transport components through vesicle cargo proteins to control the targeting of receptors to specific cellular compartments after internalization (264, 265).

## CXCR2 is essential for GPCRs internalization and neutrophil migration

Currently, 46 human chemokines are known to activate 20 chemokine receptors (266, 267) and another four atypical chemokine receptors (ACKRs) (268) (**Table 1**). CXCR2 is a receptor for eight CXC chemokines (CXCL1-8) (269, 270), which can drive innate immune cells to infiltrate remote organs in sepsis (127, 271, 272) and seems to have a synergistic effect with lipid signaling. It can make neutrophils move from random movement to directed migration, thus forming local cell clusters (176, 188). Non-survivors of sepsis showed higher CXCR2 expression on neutrophils and more neutrophil migration dysfunction than survivors (273). CXCR2 is internalized faster than CXCR1 but takes longer to be recycled (274). Studies have shown that reverse-migrating neutrophils have reduced CXCR1 expression (275). CXCR1 desensitization in zebrafish is associated with neutrophil diffusion to sites far from the wound, and this transition involves CXCR2 signaling (189). GRK2 controls the internalization of CXCR2 while desensitized LTB4R1 remains in the plasma membrane (38), suggesting that CXCR2 but not LTB4R1 is involved in receptor internalization.

In addition, CXCR2 sometimes dimerizes chemokine receptors, forming homologous dimers through the Ala106-Lys163 region even in the absence of ligands (276). When neutrophils express both CXCR1 and CXCR2, they may also form heterodimers (277). As a ligand of the two receptors, CXCL8 can deactivate the source dimer and stabilize the homologous dimer to promote the internalization of the receptor (278). It can be seen that the activation of different ligands leads to the selective expression of GPCRs dimerization, thus regulating receptor desensitization, which may be one of the ways to fine-regulate neutrophil chemotaxis, but the specific mechanism is still unclear.

The C-terminal of CXCR2 contains a leucine-rich domain (325-329aa), the LLKIL motif (279). CXCR2 binds to a variety of proteins through this LLKIL motif, including LIM and SH3 protein 1 (LASP-1), Hsc70 interacting protein (Hip), and AP-2 (279, 280). The pathway involved in the regulation of LASP-1 affects cytoskeletal rearrangement and thus neutrophil migration (281). The Hsc70 complex formed by the binding of CXCR2 to the Hip is related to the internalization of CXCR2 (282). By binding to membrane phosphatidylinositol (PI), AP-2 is docked in the plasma membrane and causes microtubule acetylation, which is essential for the targeted movement of neutrophils (283). GRK2-phosphorylated CXCR2 can also internalize the receptor by binding β-arrestin independently of clathrin and AP-2, but this is dependent on Lys327 ubiquitination on CXCR2 (284).

CXCR2 is induced on the surface of neutrophils in a TLR2 or TLR4-dependent manner in patients with sepsis and mediates neutrophil infiltration into important organs such as the lungs, hearts, and kidneys (127). TLRs, a homolog of the Drosophila protein Toll, are a kind of PRRs that control innate immunity and contribute to the inflammatory response of DAMPs. TLRs currently have 11 forms, among which TLR4 is the signal transduction receptor of LPS (285). High mobility group box 1 (HMGB1) acts as a DAMP that interacts with TLR9 to regulate the neutrophil NET formation, thereby mediating inflammatory tissue damage (286). The activation of TLRs or PI3K on activated neutrophils can induce the expression of TNF-α and iNOS. During sepsis, iNOS inhibited the expression of selectin, integrin, and cell adhesion factor -1 and promoted the production of NO, which can not only dilate pulmonary vessels and increase vascular permeability to aggravate pulmonary vascular injury but also reduce heme oxygenase-1 to inhibit neutrophil rolling and adhesion (287–290). TLRs, TNF-α, and NO can all lead to the up-regulation of GRK2, which can be used as a negative feedback mechanism to induce CXCR2 desensitization (272, 291–293). IL-33 inhibits TLR-mediated up-regulation of GRK2 expression and CXCR2 internalization by binding to its heterodimer receptor complex signal transducer 2(ST2), thereby reducing neutrophil migration dysfunction and enhancing their recruitment to the infection sites (140, 237, 294).

However, experiments showed that CXCR2 desensitization controlled by GRK2 could not prevent the migration of neutrophils to the aggregation site after GRK2 depletion, despite the desensitization resistance, and its bacterial clearance ability was impaired, indicating that the internalization of CXCR2 was not the only factor leading to the excessive migration of neutrophils (176). In addition, miRNA may also affect CXCR2 activity. MiR-K12-3, a miRNA-125 from Kaposi sarcoma-associated herpes virus (KSHV) (295), can desensitize and internalize CXCR2 by directly reducing GRK2 expression and phosphorylating GPCRs (237). A fungus containing miRNA-1321 and miRNA-3188 expressed *in vitro* even directly reduced the microRNA expressed by CXCR2 (296).

## Potential therapeutic targets for septic ARDS

The lungs are the most common source of sepsis, accounting for 64%, which easily leads to the occurrence of ARDS (297). There are currently no specific drugs for sepsis, and the only drug approved for severe sepsis, recombinant Human (rh) APC, has been shown not significantly reduced sepsis mortality (298, 299). Similarly, many pharmacological therapies have been used to treat ARDS, including aspirin, β-2 agonists, statins, and corticosteroids, but none of them have a significant effect (300). Mechanistically, NETs are involved in the progression of ARDS induced by sepsis, and inhibition of neutrophil NETs formation may be a potential target to prevent ARDS. Aspirin inhibits neutrophil migration and thromboxane-dependent NETs formation by inhibiting platelet-neutrophil interactions (301). High-dose intravenous vitamin C (HDIVC) improves ARDS by reducing the occurrence of NETosis and the shedding of the vascular endothelial glycocalyx by down-regulating the expression of cell-free DNA in neutrophils and proteoglycan syndecan-1 in vascular endothelial cells (302). α1-antitrypsin (AAT), as a protease inhibitor, especially a NE inhibitor, inhibits neutrophil migration and NETs formation by binding to IL-8 to inhibit CXCR1/CXCL8 signaling axis (303). Sivelestat (ONO-5046), another selective NE inhibitor, also improved neutrophil-mediated vascular endothelial injury and increased vascular permeability (304). Moreover, zinc-dependent histone deacetylases (HDAC) mediate the deacetylation of histone H3, which is a necessary step to allow histone citrullination and NETs formation (305). Ricolinostat (ACY-1215), an HDAC6 inhibitor currently in Phase II clinical trials, can improve lung function by inhibiting NETosis (305). In addition, glycyrrhizin, the active component of traditional Chinese medicine, reduces neutrophil NETs formation in sepsis by inhibiting the activation of the HMGB1/TLR9/MyD88 (myeloid differentiation primary response 88) pathway (286). Inhibitors of protein tyrosine phosphatase-1B (PTP1B) reduce neutrophil chemotaxis and NETs formation by inhibiting the PI3γ/AKT/mTOR pathway downstream of CXCR4 signaling (306). Interestingly, administration of DNase-I-coated melanin-like nanospheres (DNase-I pMNSs) mitigates sepsis-associated NETosis, thereby preventing the further progression of ARDS (307). The oleic acid-based nanosystems dose-dependently inhibited the production of ROS, O_2_^-^, and NE by activated neutrophils, n, thereby reducing NETs formation, and larger nanocarriers showed greater efficiency in improving ARDS (308). The drugs coated by lipid nanocarriers have the characteristics of prolonging circulatory half-life, increasing drug stability, and enhancing focus targeting ability (309), and are especially suitable for directional lung delivery in sepsis (310). The pulmonary capillary network enables larger nanoparticles to deposit in the lung (311), where anionic nanoparticles are well tolerated (312), which may also be a new route for treating septic ARDS.

One of the signs of ARDS in patients with sepsis is increased pulmonary microvascular endothelial permeability and abnormal accumulation of neutrophils. Lung endothelial barrier damage is mediated by cell contact breakdown and actin remodeling, and the accumulation of neutrophils in the lungs is mainly driven by cytoskeletal rearrangement (155). In the early stage of sepsis, excessive inflammatory responses activate neutrophils and promote their migration to the lungs. Subsequent neutrophil sequestration in the lungs leads to abnormal tissue perfusion, which in turn leads to pulmonary capillary microcirculation disturbance. Then, neutrophils with delayed apoptosis cause lung tissue damage through the release of ROS, protease, NETs, etc., eventually leading to the occurrence of severe hypoxemia and pulmonary edema. Studies showed that the peripheral miRNA may be the key epigenetic factor of endothelial activation and neutrophil dysfunction in ARDS. MiR50-887-3 expression increased on the endothelial cells of septic patients with ARDS, promoting neutrophil trans-endothelial migration by increasing VCAM-1, TLR2, CCL5, and CXCL10 expression (313). Inhibition of miR50-887-3 expression on vascular endothelium may be a potential target for septic ARDS. Sphingosine-1-phosphate (S1P) in plasma, a lipid recognized by GPCRs (S1Pr1, S1Pr2, S1Pr3) on endothelial cells, regulates neutrophil leakage triggered by lung inflammation in mice (314). As an analog of S1P, FTY720 and its analog FTY720 s-phosphonate (Tys) enhance the barrier protection function of lung endothelial cells by activating S1Pr1 (315, 316). The binding of S1P to S1Pr1 induces actin cytoskeletal recombination, adhesion junction assembly, and VE-cadherin localization to intercellular contact areas, but S1Pr1 also undergoes time-dependent desensitization (317). Fingolimod, an S1Pr agonist with a bias toward receptor internalization and degradation, is effective in treating multiple sclerosis (318–320) and may be used to control neutrophil aggregation. In addition, the sweet taste receptor T1R3 is also a kind of G-protein-coupled receptor. Activated T1R3 may protect the lung endothelial barrier by eliminating Src/P21-activated kinase (PAK)/p110αPI3K-mediated cell contact breakdown and Src/myosin light chain 2 (MLC2)/heat shock protein 27 (HSP27) -mediated actin remodeling (321). *In vitro* studies have shown that exogenous prostaglandin F2α (PGF2α) promotes neutrophil migration in endometrial carcinoma by up-regulating CXCR2/CXCL1 expression through PGF2α receptors (also known as FP receptors, a type of GPCRs) (322, 323). However, FP receptor antagonist AL8810 promotes neutrophil migration to BAFL by increasing adhesion molecules expression on the vascular endothelium such as ICAM-1 and E-selectin, while decreasing the gene expression of alveolar surfactant protein and aggravating pulmonary edema (324). FP receptor agonists may have the potential to treat septic ARDS, but this needs further study. The administration of ligands binding to GPCRs on endothelial cells to activate intracellular signal transduction pathways mediates cytoskeletal recombination to inhibit neutrophil aggregation to the lung, which may be a new therapeutic target for lung endothelial cell protection in septic ARDS.

On the other hand, modifying the chemotaxis of neutrophils by regulating the internalization of GPCRs may improve the inflammatory response in the lungs during sepsis. Barbadin is a novel inhibitor that blocks the interaction of β-arrestin and AP-2, reducing receptor desensitization by inhibiting clathrin-mediated internalization of GPCRs, thus allowing neutrophils to continuously respond to external stimuli (325, 326). Furthermore, LMBD1, the first protein was shown to be involved in the regulation of insulin receptor (IR) internalization (327), is hypothesized to contain nine transmembrane domains that subcellular localize in multiple membrane-bound organelles, including lysosomes and plasma membranes (328). LMBD1 can interact with AP-2 and selectively participate in clathrin-mediated IR endocytosis (327). When LMBD1 is deficient, IR is retained in the plasma membrane, suggesting that LMBRD1 may be an important molecule mediating GPCRs internalization. In addition, administration of the glycolytic inhibitor 2-deoxyglucose (2-DG) restored the ability of neutrophils to cluster to infected abdominal lesions in sepsis mice, partially reversed glycolytic-induced neutrophils migration dysfunction and down-regulated CXCR2 expression, suggesting that 2-DG may be a potential therapeutic strategy (329).

## Conclusion

ARDS is an important cause of acute respiratory failure, which is usually associated with multiple organ failure, and sepsis is one of its common causes (330). The incidence of sepsis-induced ARDS remains high despite the clinical efficacy of antibiotic therapy during infection (1). The lung is usually the first target organ attacked by multiple organ dysfunction in sepsis (331, 332). The main pathophysiological changes are the destruction of the alveoli-capillary barrier (333), which leads to increased permeability of pulmonary blood vessels, increased migration of neutrophils to the lungs, and accumulation of protein-rich edema fluid in the alveoli, thus causing tissue and organ damage (334). Due to the difficulty in balancing the specificity and sensitivity of currently available biomarkers, finding new targeted therapeutic markers is still crucial to prevent ARDS in patients with sepsis.

The excessive inflammatory response shown in the early stage of sepsis leads to a large accumulation of neutrophils in the lungs, excessive activation, and delayed apoptosis, which is an important cause of acute lung injury or acute respiratory distress syndrome. In sepsis, the impaired migration of neutrophils to the pulmonary tissues may be due to excessive binding to vascular endothelial cells during initial adhesion, followed by reduced chemotactic reactivity due to GPCR desensitization and internalization, resulting in strong infiltration of these cells into the infected lung tissue. Therefore, the ideal treatment strategy for septic ARDS is to reduce the accumulation of neutrophils in the lungs without impairing its microbial clearance and regenerative capacity (335, 336) Cytoskeleton-regulated subcellular localization and conformational regulation such as phosphorylation and dimerization are important mechanisms of GPCRs desensitization. According to the ligand-bias and phosphoryl-barcoding hypothesis, different ligands preferentially promote different downstream GPCR pathways, so it is important to develop allosteric regulators to precisely control the effects of activated neutrophils. At present, the main treatment method for septic ARDS is still a lung-supporting ventilation strategy (337). Targeting GPCRs to regulate neutrophil chemotaxis to improve pulmonary inflammation and microcirculation may be a promising field for the treatment of septic ARDS. On the other hand, targeting the interaction between neutrophils and GPCRs on pulmonary vascular endothelial cells may reduce the over-aggregation of neutrophils in the lungs, which may also be an effective strategy. Although other pathways inhibit neutrophil migration, such as activating proteins C (338), ROS (339), and NETs (340), this review focuses on the physiological mechanism of how GPCRs expressed in sepsis patients cause ARDS by influencing neutrophil migration to improve the prognosis of patients and prevent the occurrence of irreversible MODS, providing more valuable reference targets for the development of new therapies.

## Author contributions

YW, C-LZ, and J-FW reviewed the literature, wrote drafts of the manuscript, and prepared figures. X-MD, PL, QL, H-RL, and C-MY helped in the evaluation of the literature and the submission of the manuscript. J-FW and X-MD designed and supervised the work. All authors contributed to the review and approved the submitted version.
